# Migration of Cardiac Cells: Hot Topics that have not Attracted Clinical Attention

**DOI:** 10.7150/ijms.130458

**Published:** 2026-07-11

**Authors:** Jie Ma, Yaping Xu, Yali Wang, Zhikun Guo

**Affiliations:** 1 Department of Human Anatomy & Histoembryology, School of Basic Medical Sciences, Henan Medical University, Xinxiang 453003 P.R. China.; 2 Henan Key Laboratory of cardiac remodeling and transplantation, Zhengzhou Seventh People's Hospital, Zhengzhou 45300 P.R. China.; 3 Henan Key Laboratory of Medical Tissue Regeneration, Henan Medical University, Xinxiang 453003 P.R. China.; 4 Department of Physiology & Pathophysiology, School of Basic Medical Sciences, Henan Medical University, Xinxiang 453003 P.R. China.

**Keywords:** cytokines, cell migration, myocardial infarction, heart failure

## Abstract

Cell migration plays a critical role in various physiological and pathological processes, particularly in the development and repair of cardiac diseases. It is a complex, multi-step process involving different mechanisms and cell types. This review summarizes the roles of cardiac cell migration in cardiac immune responses, myocardial regeneration, and chronic inflammation. Specifically, we explore how cardiac cells promote repair and regeneration following myocardial injury through migration. In addition, the article reviews the impact of cell migration on processes such as angiogenesis and fibrosis, which are crucial for myocardial repair and pathological states. Due to the complexity of intercellular interactions, signaling pathways, and microenvironmental factors, understanding cardiac cell migration remains a significant challenge. Further research into these migratory mechanisms will help uncover their pathological roles in cardiac diseases and provide new therapeutic approaches for cardiac repair and regeneration. This review also highlights the migratory mechanisms of major cardiac cell types and examines the regulatory roles of chemokines in these processes. Furthermore, we discuss how cardiac cells engage in localized migration under different disease conditions, thereby contributing to the heart's self-repair.

## Introduction

Cell migration, also known as cell crawling or cell movement, refers to the displacement of cells in response to migration signals or stimuli from specific substance gradients. This process is crucial for the establishment and maintenance of normal tissue structure in multicellular organisms and plays a central role in maintaining homeostasis, disease progression, and developmental biology such as development, wound healing, tissue homeostasis, and certain types of cancer metastasis 1-4. As a hallmark feature of living cells, cell migration encompasses both physiological and pathological migration 5. Based on the quantitative behaviors of cell migration, it can be categorized into collective migration and single-cell migration. Collective migration primarily occurs during embryonic development 6-8, while single-cell migration is typically observed in isolated cells, such as leukocytes during immune responses or fibroblasts in connective tissue 1 Moreover, single-cell migration is often studied in *in vitro* experiments. As a ubiquitous biological phenomenon, cell migration can be observed in almost all organs. However, the migration patterns, mechanisms, and outcomes vary significantly among different cell types 9.

Cardiac cell migration plays a critical role not only in normal heart development and repair but also in the treatment and regeneration of cardiac diseases. For example, after myocardial infarction, the migration of cardiac cells promotes the repair and regeneration of damaged myocardium, offering potential new avenues for clinical therapy 10. However, the understanding of the mechanisms underlying cardiac cell migration remains limited and faces significant challenges, particularly regarding the complex migratory behaviors under pathological conditions of the heart. Therefore, exploring the principles and mechanisms of cardiac cell migration is of great importance for advancing cardiac repair and regeneration.

This article will focus on research related to cardiac cell migration. It will first review the fundamental mechanisms of cardiac cell migration, then discuss the migratory behaviors of cardiac cells under different pathological conditions, and finally analyze the potential applications of cell migration in cardiac repair.

## The mechanisms of cardiac cell migration

The cells of the heart include resident cells and those derived from the bloodstream, known as cardiac circulatory cells. Resident cells encompass cardiomyocytes, fibroblasts, vascular smooth muscle cells, and endothelial cells. Under both physiological and pathological conditions, resident cells migrate freely among the intercellular substances to maintain the homeostasis of the myocardial microenvironment and facilitate the repair of diseased myocardium. Conversely, cells originating from the bloodstream must traverse the vascular wall and basement membrane to access myocardial tissue. The homing of stem cells involves the directed migration from the bloodstream, across capillary walls, into damaged myocardium, a process mediated by chemokines. Using the cardiac targeting homing of mesenchymal stem cells (MSCs) as an example, the migration of cells from intravascular spaces to myocardial tissue primarily comprises the following five steps 11.

The migration mechanisms of fibroblasts and MSCs exhibit both similarities and differences. *In vitro*, fibroblasts migrate at varying speeds and exhibit diverse morphologies. In cell culture, fibroblasts move slowly, with an average speed of less than 1 μm/min, and frequently change direction. The migration process of fibroblasts involves the protrusion and subsequent adhesion at the leading edge, the development of contractile forces between the leading and trailing edges, and the eventual release of trailing adhesions due to applied tension and/or enzymatic activity. Cell retraction generates an excess dorsal surface, which sustains the protrusion in a process known as retraction-induced spreading. This is considered a classic perspective on fibroblast migration 12.

When tissues are damaged *in vivo*, numerous growth factors are produced, including epidermal growth factor (EGF) and platelet-derived growth factor (PDGF). Stimulation by these growth factors can increase the migration speed of individual fibroblasts by up to threefold and alter the direction of their migration. Vimentin, a type III intermediate filament protein expressed in mesenchymal cells, along with microtubules and microfilaments, constitutes the cytoskeleton. Vimentin expression contributes to cellular mechanoprotection and serves as a widely recognized marker of fibroblasts and epithelial-mesenchymal transition 13. Vimentin significantly influences the adhesion and migration of fibroblasts and myofibroblasts on collagen 14. The binding of cells to the extracellular matrix (ECM) promotes the association of vimentin with the cytoplasmic domains of adhesion receptors, such as integrins. Following initial adhesion, cell-generated myosin-dependent forces and signals that impact vimentin structure can subsequently affect cell migration 13.

There is limited literature on the migration of adult human cardiac myocytes. In the process of myocardial regeneration in some lower animals, the migration of myocardial cells can be observed. The migrating myocardial cells are characterized by cell protrusions or projections. After 2 days of myocardial tissue resection, most of the migrating myocardial cells move along the boundaries of neovascularization. 69% of myocardial cell protrusions are within a range of 15 μ m from the nearest blood vessel, which represents the average diameter of two cell nuclei. The average distance between myocardial cell protrusions and their nearest blood vessels is 12.4 ± 0.9 μm 15. The mechanisms of neovascularisation and cardiomyocyte migration after apicoectomy in neonatal mice remain to be elucidated. A study using zebrafish ventricular amputation demonstrated that epicardium-derived stromal-derived factor (SDF)-1α induces cardiomyocyte migration. Notably, the SDF-1α receptor, CXCR4b, is expressed in cardiomyocytes but not in endothelial cells. Furthermore, blockade of the CXCR4b receptor results in defective cardiomyocyte migration but does not affect endothelial cells. During the formation of zebrafish coronary arteries, SDF-1β guides endothelial cells. Therefore, the distance between migrating myocardial cells and blood vessels is very close, which may be due to the presence of a common chemotactic inducer, such as SDF-1α, that stimulate endothelial and myocardial cell migration 16, 17. Due to the expression of CXCR4 in both myocardial cells and arterial endothelial cells, SDF-1α derived from arterial endothelium may guide a series of vascular sprouting, arterial generation, and myocardial cell migration after myocardial injury 18.

**Figure 1:**
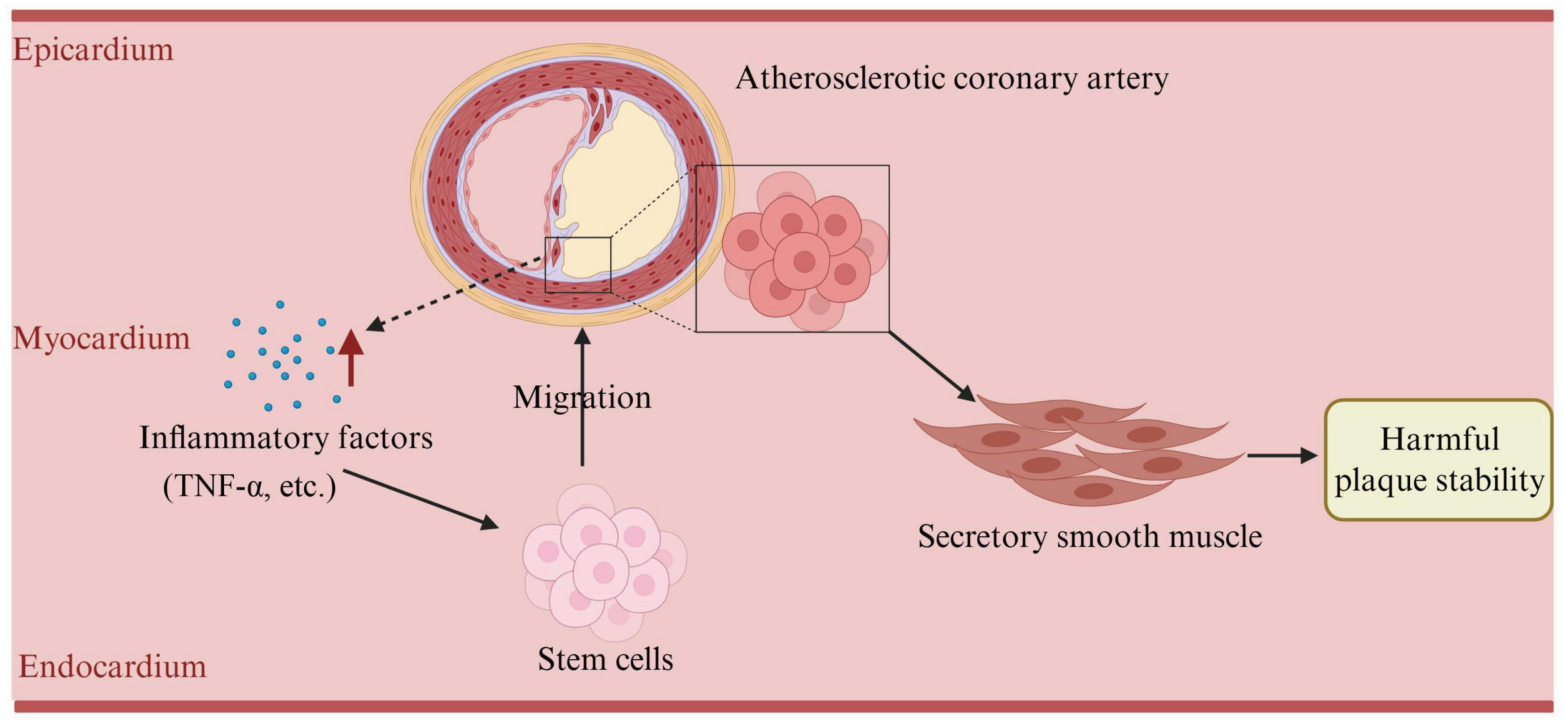
Schematic diagram of migration of myocardial stem cells to the atherosclerotic area of coronary arteries (This figure is drawn based on our research results).

Recently, we have proved that TNF-α, as a chemokine, can attract Nanog and Sca-1 positive cells (a kind of stem cells) in myocardial tissue to migrate to the coronary atherosclerosis area, and these migrated cells differentiate into secretory smooth muscle around the lesion. This result suggests that under the guidance of chemokines, cardiac stem cells converged to the focus and only changed the structure of the arterial wall, which did not play a significant role in the treatment of atherosclerotic plaque (Figure 1).

In summary, numerous factors promote cell migration, including various inflammatory mediators, growth factors, alterations in the intercellular matrix, and other environmental cues. These factors and the surrounding microenvironment influence target cells, inducing changes in cell morphology and ultimately directing their movement.

## Cell chemokines and cell migration

Chemokines are a group of small molecules specific to vertebrates that regulate cell migration and behavior in diverse contexts. So far, approximately 50 chemokines have been identified in humans, which bind to 18 different chemokine receptors. These receptors are members of the seven-transmembrane receptor family. Chemokines and their receptors directly affect the migration of endothelial cells, thereby influencing angiogenesis 19. Based on molecular structure, chemokines can be categorized into four groups: XC, CC, CXC, and CX3C. Furthermore, chemokines can be classified into two functional categories: inflammatory chemokines and homeostatic chemokines.

### Inflammatory chemokines and cardiomyocyte migration

Chemokines and their receptors control the migration and residence of all immune cells. Some chemokines are considered pro-inflammatory, and their release can be induced during an immune response at a site of infection. Generally, CXC chemokines are chemotactic for neutrophils, while CC chemokines attract monocytes and a subset of lymphocytes 20. Damage and disease in the heart can prompt the migration of various cells, including fibroblasts, within myocardial tissue. Chemokines are involved in the migration of cardiac fibroblasts. The phenotype and function of cardiac fibroblasts undergo significant changes after myocardial infarction. In the early stages of infarct healing, fibroblasts transform into pro-inflammatory cells. They activate the inflammasome and produce cytokines, chemokines, and proteases. The reduction of the inflammatory infiltrate in the infarcted area is linked with fibroblast migration, proliferation, synthesis of matrix proteins, and conversion into myofibroblasts 21. In the healing of infarcts, the CXC chemokine Interferon-γ Inducible Protein (IP)-10 plays an anti-fibrotic role and inhibits fibroblast migration 22. The upregulation of chemokine SDF-1α and its receptor CXCR4 after myocardial infarction may play a crucial role in the homing and migration of stem cells 23. In heart failure, Th1 and Th17 cells contribute to persistent pathological chronic inflammation, cell migration, and a specific pathological phenotype of monocytes through inflammatory factors 24. Myocardial ischemia-reperfusion (MIR) injury is characterized by a rapid increase in cytokines and chemokines, along with the infiltration of inflammatory cells. Liu et al. utilized Sparstolonin B (SsnB) in experiments on hypoxia reoxygenation injury and the transwell migration test of myocardial cells. Their findings revealed that SsnB significantly reduced the migration of mouse macrophages to myocardial cells injured by hypoxia reoxygenation. The underlying mechanism involves SsnB markedly mitigating myocardial inflammation induced by hypoxia reoxygenation, achieved by inhibiting the ERK1/2 and JNK signaling pathways 25. Additionally, Rienks' team demonstrated that recombinant Sema3A protein impacts the pro-inflammatory status of cultured bone marrow-derived macrophages and can delay the migration of monocytes to the myocardium 26. Ingason et al. discovered that both endothelial cells and myocardial cells migrated to the injured site after resecting the cardiac apex in mice. However, their migration sequence differed. Endothelial cells migrated to the apical thrombus early, initiating the development of functional arteries. Subsequently, they grew inward and migrated before myocardial cells migration 15. *In vitro* experiments demonstrate that hypoxic cardiomyocytes produce inflammatory factors such as TNF-α, IL-1β, IL-6, and TGF-β. These metabolites from early hypoxic cardiomyocytes can induce the migration of cardiac fibroblasts. Specifically, TNF-α and IL-1β may serve as the initial chemotactic inducers 27. Hence, ample experimental evidence confirms the pivotal role of inflammatory chemokines in promoting cell migration.

### Homeostatic chemokines and cell migration

Homeostatic chemokines play a crucial role in controlling the migration and activation of stem and progenitor cells during vasculogenesis and organ development. They also regulate the niches of peripherally directed progenitor cells for tissue renewal. These biological functions support angiogenesis and wound healing, including the recruitment of endothelial and other progenitor cells from the bone marrow 28. The chemokine CCL21 is independently associated with the prognosis of acute coronary syndrome, making it a promising biomarker for further research in patients 29. Homeostatic chemokines contribute to B-CLL resistance to cell death by inactivating the transcription factor FOXO3a. This factor is regarded as a novel therapeutic target for hematopoietic malignancies 30. Homeostatic chemokines respond to the chemotaxis induced by various inflammatory factors through chemokine receptors expressed on MSCs, including C-C Motif Chemokine receptors (CCR) 1-11, CXCR1-7, and CX3CR1. These chemokine/chemokine receptors play a pivotal role in directing the migration of MSCs to tissues 31.

Fibroblasts constitute approximately 70% of the cells in heart tissue. Regardless of physiological or pathological conditions, cardiac fibroblasts continually migrate and traverse through the intercellular substance. Historically, literature on fibroblast migration primarily focused on wound healing or *in vitro* cell experiments 32-34. Studies involving *in vivo* myocardial fibroblasts mostly pertained to myocardial fibrosis 35, 36. There is limited research on the migration of cardiac fibroblasts between tissues. *In vitro* studies have shown that cardiac fibroblasts migrate at varying speeds and shapes compared to single fibroblasts in cell culture. It is well-established that numerous growth factors present at wound sites serve as mitogens or chemokines for fibroblasts, including EGF and PDGF. Stimulation by these growth factors can increase the migration speed of single fibroblasts by up to three times and alter the direction of cell migration 37. In recent years, research on cytokines and myocardial cell migration has been gaining attention. Studies have revealed that chemerin-9 stimulates migration, possibly through the ROS-dependent activation of Akt and ERK via CMKLR1 in cardiac fibroblasts. These findings suggest that chemerin may play a significant role in the pathogenesis of cardiac diseases 38. Circ-LAS1L regulates cardiac fibroblast activation, growth, and migration through the miR-125b/SFRP5 pathway 39. Additionally, miR-590-3p regulates the proliferation, migration, and collagen synthesis of cardiac fibroblasts by targeting ZEB1^40^. Hence, in response to various cytokines, fibroblasts within myocardial tissue exhibit collective migration through the intercellular substance, enabling them to migrate to injured or functional sites. This collective migration mechanism ensures the homeostasis of myocardial tissue.

**Figure 2:**
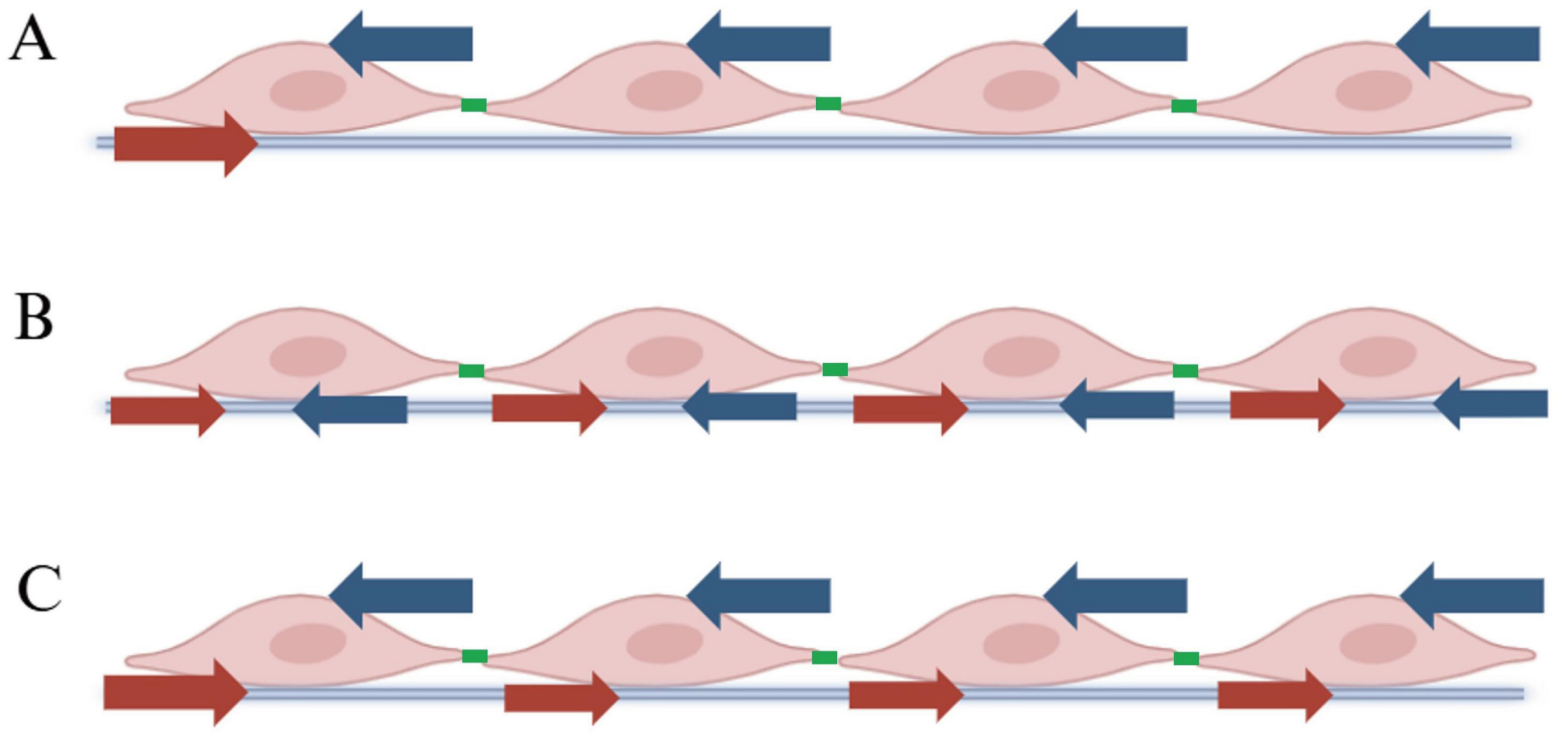
Patterns of force generation and transmission in an epithelial cell sheet (adapted from reference 12). (A) An active leader cell generates forces at the leading edge and transmits these forces to follower cells via cell-cell junctions. The endothelial cells of the myocardium possess this force transmission mode. (B) Each cell within the monolayer generates its own contractile forces. Forces are balanced locally, resulting in no force transmission through cell-cell junctions. Early developing myocardial cells exhibit this migration pattern. (C) Tug-of-war force generation and transmission occurs, where local tractions generated by each cell are transmitted through cell-cell junctions to create a global gradient of tensile stress. Both fibroblasts and endothelial cells in the myocardium exhibit this migration pattern.

Compared to the mechanisms driving single-cell migration, collective cell migration remains less understood. However, it shares similarities with single-cell migration, including protrusion, polarization, contraction, and adhesion to the surrounding matrix. The most extensively studied mode of collective cell migration involves the advancement of 2D epithelial sheets over a basement membrane. These moving cell sheets typically consist of a large number of cells that maintain cohesion as they invade open spaces or surrounding tissues. The dorsal closure process in drosophila exemplifies collective cell migration driven by at least three mechanisms: (1) Active migration of epidermal cells, characterized by dynamic filopodia extension at the anterior edge; (2) Periodic contraction of the amniotic serosa; (3) Pulling of a supracellular actin cable at the leading edge 41, 42. These examples illustrate that even in the simplest instances of collective migration, the movement of a population is not solely driven by the independent actions of individual cells within the migrating group. Instead, it is influenced by the synergistic interactions between cellular mechanisms and the surrounding tissues. In terms of mechanical transmission, epithelial cell migration also presents three types of force transmission (Figure 2).

According to the functional state of cell migration, it can be divided into physiological cell migration and pathological cell migration. The former mainly occurs during the processes of embryonic development, tissue repair, immune cell homing, and angiogenesis; the latter mainly occurs in the occurrence and development of tumor metastasis, chronic inflammation, atherosclerosis, fibrosis, etc. Physiological cell migration is controlled, directed, limited, and reversible, while pathological cell migration is uncontrolled, invasive, persistent, and irreversible. From a mechanistic perspective, there are also many differences between physiological and pathological cell migration. For example, physiological cell migration has more precise navigation and orderly cell polarization, while pathological cell migration is characterized by uncontrolled cell signal regulation and disrupted cell polarization. In short, physiological migration is a controlled process of “precise navigation, orderly collaboration, and task orientation”; pathological migration is an infinite expansion process characterized by signal loss control, polarity disorder, adhesion disorder, and matrix invasion.

### The dual/context-dependent nature of chemokines

Numerous chemokines can exhibit entirely opposing biological effects (pro-inflammatory/anti-inflammatory, pro-tumor/anti-tumor, pro-repair/pro-damage) under different microenvironments, cell types, concentrations, time points, or disease stages. Examples include IFN-γ, IL-10, TGF-β, IL-6, TNF-α, IL-2, and SDF-1α. These chemokines serve as a double-edged sword in immunotherapy: both blockade and activation may yield positive or negative outcomes, necessitating precise spatiotemporal regulation (dose, localization, timing, combination) to achieve therapeutic benefits. For instance, chemokines participate in angiogenesis with both anti-angiogenic and pro-angiogenic factors, while also interacting with other angiogenic molecules^43^.

SDF-1 α (stromal cell-derived factor 1 α, also known as CXCL12) is a member of the CXC chemokine family, with strong dual/scenario dependent effects. SDF-1α is the “navigation lighthouse” for tissue repair and also an “accomplice” to tumors/fibrosis, with the ultimate effect entirely dependent on concentration, receptors, microenvironment, and disease stage. In terms of anti-tumor effects, SDF-1α promotes the proliferation of tumor cells and overcomes the cytotoxic effects of chemotherapy. CXCR4 antagonists (such as Plexafoc) can block cell metastasis and enhance the efficacy of immunotherapy 44. Blocking the SDF-1α/CXCR4 axis provides protection against myocardial ischemia-reperfusion injury and has anti fibrotic effects 45. *In vitro* experiments show that SDF-1α can significantly enhance the migration, tube formation, and monocyte adhesion of human umbilical vein endothelial cells, demonstrating strong pro-angiogenic activity. *In vivo* experiments show SDF-1α can significantly enhance angiogenesis 46. The effective synergy between ICA and SDF-1α can support *in vivo* cartilage formation 47.

The SDF-1α/CXCR4 axis contributes to myocardial protection after myocardial infarction by recruiting endogenous stem cells into ischemic tissue. However, it can also recruit too many inflammatory macrophages, exacerbating myocardial damage. More seriously, the increase in inflammation leads to abnormal electrical coupling of myocardial cells, resulting in uneven ventricular conduction and slower conduction velocity. Therefore, it is highly desirable to selectively recruit stem cells while also blocking inflammation 48. After myocardial infarction, cytokines released by macrophages (such as oncostatin M) are not only used to uptake apoptotic or damaged myocardial cells, but also promote myocardial cell proliferation and regeneration. After myocardial infarction, macrophages can also achieve complete regeneration of myocardial tissue by coordinating inflammation and matrix deposition to reshape myocardial cell proliferation 49, 50.

## Cell migration in several common heart diseases

In various heart diseases, the migration of cardiomyocytes, fibroblasts, and immune cells within cardiac tissue plays a crucial role. Whether under physiological or pathological conditions, this migration is essential for maintaining the microenvironmental balance of cardiac structure and function. The characteristics and mechanisms of cell migration vary across different heart diseases. For example, in myocardial infarction, fibroblasts migrate to the injured site to participate in scar formation, while immune cells help clear necrotic tissue. In heart failure and dilated cardiomyopathy, fibroblast migration contributes to myocardial fibrosis, which affects cardiac function. In hypertrophic cardiomyopathy, excessive cell migration may exacerbate myocardial stiffness. A deeper understanding of cell migration characteristics in different heart diseases can aid in developing new therapeutic strategies and promoting myocardial repair.

### Myocardial infarction and cell migration

The repair process after myocardial infarction can be divided into several key stages, which are interwoven within the inflammatory, proliferative, and maturation phases 51. During this process, cell migration is precisely regulated by various signaling factors. Different types of cells interact through these factors to collectively promote myocardial repair, ultimately contributing to the restoration of cardiac structure and function (Table 1). Cell death triggers a sterile inflammatory response through endogenous damage-associated molecular patterns (DAMPs), followed by the cascade release of chemokines and pro-inflammatory cytokines. This activates neutronphils, monocytes, and macrophages, prompting their migration to the infarcted area to clear necrotic cells. Approximately 10 days later, fibroblasts proliferate and migrate to the infarcted region under the guidance of inflammatory signals, contributing to scar formation. Simultaneously, under the action of signaling pathways such as VEGF and Notch, endothelial cells and endothelial progenitor cells migrate to the damaged area, promoting blood supply recovery and the formation of new blood vessels. We discovered ten years ago that after myocardial infarction in rats, myocardial stem cells, such as Nanog-positive cells, quickly gather in the border area of the infarction and participate in myocardial repair 52. As the repair process progresses into the maturation phase, extracellular matrix remodeling and endothelial cell migration during angiogenesis play a crucial role in neovascularization and the recovery of cardiac function.

#### Cardiomyocyte migration

The migration of myocardial cells mainly occurs during embryonic development and after myocardial injury. There is limited literature on whether positional movement of myocardial cells occurs in normal myocardial tissue. After birth, mammalian myocardial cells retain a certain degree of proliferative ability, but they soon lose their mitotic capability. However, decreased mitochondrial function and increased glucose utilization can restore the mitotic ability of adult cardiomyocytes, leading to the production of new cardiac cells. At the same time, newly formed cells undergo positional migration to meet the needs of structural adjustment 53. BMSCs-IGF-1 promotes cell proliferation, migration ability, stem cell characteristics, and has greater resistance to apoptosis under hypoxia 54. Oxygen metabolism and oxidative stress play a crucial role in regulating the proliferative ability of mammalian cardiomyocytes. Reducing oxygen metabolism in the hearts of adult mammals can induce myocardial cell cycle reentry through reduced oxidative damage, which is sufficient for functional improvement after myocardial infarction 55. After myocardial injury, both capillary endothelial cells and myocardial cells migrate to the damaged area to jointly construct myocardial regeneration, but there is a difference in the time of migration between the two. After removing the apex of the heart in newborn mice, angiogenesis in the damaged area occurs before the migration of myocardial cells. In the early stage after resection, endothelial cells migrate to the top thrombus and develop into functional arteries, followed by myocardial cells growing into this area. This provides blood supply for myocardial cells before they migrate to their destination 15.

There are many cell signals involved in myocardial cell migration. Activation of CXCL12/CXCR4, Nrg1-ErbB2/ErbB4, ERK, PI3K/Akt can all promote adult myocardial cell migration 56, 57. Under the regulation of MMP-2, the SDF-1α/CXCR4 axis and hypoxia also induce the migration of human adult cardiomyocytes 58. It should be emphasized that cell migration only focuses on the cells themselves, which is not comprehensive. The extracellular matrix of the heart plays an important role in the regeneration and recovery process. The epicardium, endocardium, and pericytes reactivate the embryonic program under extracellular matrix stimulation, leading to epithelial mesenchymal transition, cell migration, and differentiation 58.

It has been reported that the infarcted area of adult mammalian hearts contains various types of exogenous progenitor cells, which can be directly transformed into cardiomyocytes to generate new myocardium. However, these conclusions have been controversial, as subsequent studies from independent laboratories have failed to observe such results. Later, the consensus in this field gradually shifted to the fact that adult mammalian cardiac muscle undergoes low-level renewal of new cardiac muscle cells every year, and those renewed cells mainly come from the proliferation of existing cardiac muscle cells, rather than assumed progenitor cells 59. In short, there is a certain relationship between cell migration and cell proliferation. Due to the uncertain proliferation of myocardial cells, little is known about their migration, and there is still much research to be done.

#### Neutrophil migration

Neutrophils play a complex dual role after AMI and are one of the key cell types influencing post-infarction myocardial repair. Following AMI, neutrophils act as pioneer cells in the acute inflammatory response, rapidly migrating to the infarcted area and surrounding tissues, with their numbers increasing significantly 60. This migratory process is primarily driven by increased release from the bone marrow and spleen, enhanced production, and reduced apoptosis of neutrophils 61. It is finely regulated by various chemokines (such as CXCL1, CXCL2, CXCL5/CXCL8) and granulocyte-macrophage colony-stimulating factor (GM-CSF). In addition, chemokines such as CCL2, CCL3, and CCL5, along with their corresponding receptors (CCR1, CCR2, CCR5), also play critical roles in promoting the directional recruitment and activation of neutrophils 62. Studies have shown that blocking the CCL5 pathway significantly reduces neutrophil migration to the infarct zone, thereby reducing infarct size and improving cardiac function 63.

To further understand the regulatory mechanisms of neutrophil migration, some studies have found that GM-CSF is transiently expressed by cardiac fibroblasts in the early stages of AMI 64. This factor not only participates in the inflammatory response but also transmits signals to the bone marrow to mobilize and recruit neutrophils, demonstrating its dual role in amplifying inflammation and immune cell recruitment 64. Another important pathway is the Dectin-1 signaling pathway: in ischemia-reperfusion models, Dectin-1 expression is upregulated in bone marrow-derived macrophages, enhancing the expression of CXCL1 and G-CSF, thereby promoting neutrophil recruitment 65. Inhibition of Dectin-1 expression or function has been shown to improve cardiac function, suggesting its potential as a therapeutic target in AMI 65. Inhibition of Dectin-1 expression or function has been shown to improve cardiac function, suggesting its potential as a therapeutic target in AMI 51. Additionally, growth differentiation factor 15 (GDF-15) interferes with chemokine-induced integrin activation, limiting neutrophil migration to the infarct zone 66. It plays a key role in myocardial protection and animal survival, acting through TGF-β receptors I and II, revealing the complexity of inflammatory regulatory networks 66.

Neutrophil migration exhibits a double-edged effect in both time and space. In the early phase of infarction, appropriate neutrophil recruitment helps remove necrotic tissue, release remodeling signals, and create a microenvironment conducive to the entry of reparative cells. However, when this migration is excessive or dysregulated, it leads to sustained release of reactive oxygen species (ROS) and pro-inflammatory cytokines (such as TNF-α, IL-1β, and IL-6), which can induce secondary myocardial injury and pathological remodeling, ultimately resulting in deteriorated cardiac function 67. Therefore, precise regulation of the magnitude and timing of neutrophil migration is a key strategy for preventing post-AMI inflammatory imbalance and promoting myocardial repair. Recent studies have focused on targeting the CXCL8-CXCR2 signaling axis, reducing ROS production, or developing neutrophil-targeted anti-inflammatory therapies, all of which show promise in improving post-AMI myocardial healing and preventing adverse ventricular remodeling. In the future, targeted interventions on neutrophil migration pathways may open new avenues for AMI treatment.

#### Monocyte and macrophage migration

Macrophages primarily originate from circulating monocytes, which differentiate after crossing the vascular endothelium. In young mice, macrophages account for approximately 5%-10% of non-cardiomyocytes in the heart 68. Remarkably, as early as 5 minutes after MI, macrophages rapidly accumulate in the infarct region, indicating a high capacity for migration and spatial redistribution 69.

Within hours to days following MI, the number of monocytes in the heart increases significantly, mainly derived from bone marrow and splenic monocyte reservoirs. As early as 30 minutes after infarction onset, circulating monocytes begin to migrate into the injured myocardium. Subsequently, under the stimulation of angiotensin II (Ang II), splenic monocytes are mobilized into the bloodstream and recruited to the infarct zone. These monocytes not only participate in inflammation but also facilitate neutrophil recruitment. By day 1 post-MI, resident cardiac macrophages are nearly depleted and replaced by newly recruited monocyte-derived macrophages. From day 2 to day 5, these cells constitute the major immune population in the heart 70. As the reparative phase progresses, the number of infiltrating cells gradually decreases, and these newly differentiated macrophages may permanently replace the embryonically-derived resident population.

The CCR2/CCL2 signaling axis plays a central role in the proliferation and mobilization of bone marrow-derived monocytes. Upon tissue injury or infection, endothelial cells, fibroblasts, and smooth muscle cells locally produce CCL2, which activates CCR2⁺ hematopoietic progenitors to enter circulation. Simultaneously, Ang II triggers splenic monocyte release, accounting for up to 50% of all monocytes recruited to the infarct area 71. Once in the infarct zone, monocytes predominantly differentiate into M1-type macrophages, responsible for clearing necrotic and apoptotic cells. However, sustained M1 presence may prolong inflammation, promote infarct expansion, and exacerbate adverse ventricular remodeling. Therefore, regulating macrophage polarization has emerged as a promising cardioprotective strategy. For instance, RNA interference (e.g., RNAi of CCR2) targeting monocytes has been shown to inhibit their migration to the infarct site, reduce M1 macrophage infiltration, and preserve resident macrophages, representing a potential therapeutic approach 72.

Multiple inflammatory mediators regulate monocyte/macrophage migration. IL-1α and IL-1β, released by damaged cardiomyocytes, promote leukocyte recruitment. Inhibiting IL-1 signaling reduces infarct size and prevents maladaptive remodeling 73. The CCR2/CCL2 axis is a critical regulator of monocyte recruitment, and its blockade leads to smaller infarcts and improved cardiac function 74. CCL5, which recruits both neutrophils and macrophages, has also been targeted—monoclonal antibody treatment against CCL5 in non-reperfused MI models significantly reduces immune cell infiltration and infarct size 75. CXC chemokines primarily facilitate neutrophil migration during ischemia 76, while CCR9 regulates the migration of lymphocytes, dendritic cells, and monocytes/macrophages and contributes to limiting inflammation during acute MI 77.

Recent studies have identified legumain (Lgmn) as a macrophage-specific gene in the heart, with expression significantly upregulated after MI. Knockout of Lgmn enhances the infiltration of CCR2⁺ MHC-II^high^ macrophages and promotes recruitment of CCR2⁺ MHC-II^low^ monocytes, indicating a regulatory role in macrophage phenotypic balance 78. Additionally, perivascular B cells in the infarcted myocardium have been shown to upregulate MHC-II expression in nearby CCR2⁺ macrophages via paracrine signaling, promoting expansion of the CCR2⁺ MHC-II^high^ subset 79.

### Fibroblast migration

Fibroblasts are the most abundant cell type in the heart, and their functions are complex, so the literature on fibroblast migration is extensive. Fibroblasts are essential for establishing and maintaining the structural integrity of all organs. They can acquire inflammatory phenotypes to adapt immune cells to specific niches and provide migration, differentiation, and growth factors. In the heart, the balance of fibroblast activity is crucial for optimal organ function during both cardiac homeostasis and inflammation. During inflammatory perturbation, cardiac fibroblasts rapidly transition to an inflammatory state and actively communicate with infiltrating immune cells to coordinate the migration and activity of immune cells 80.

**Figure 3:**
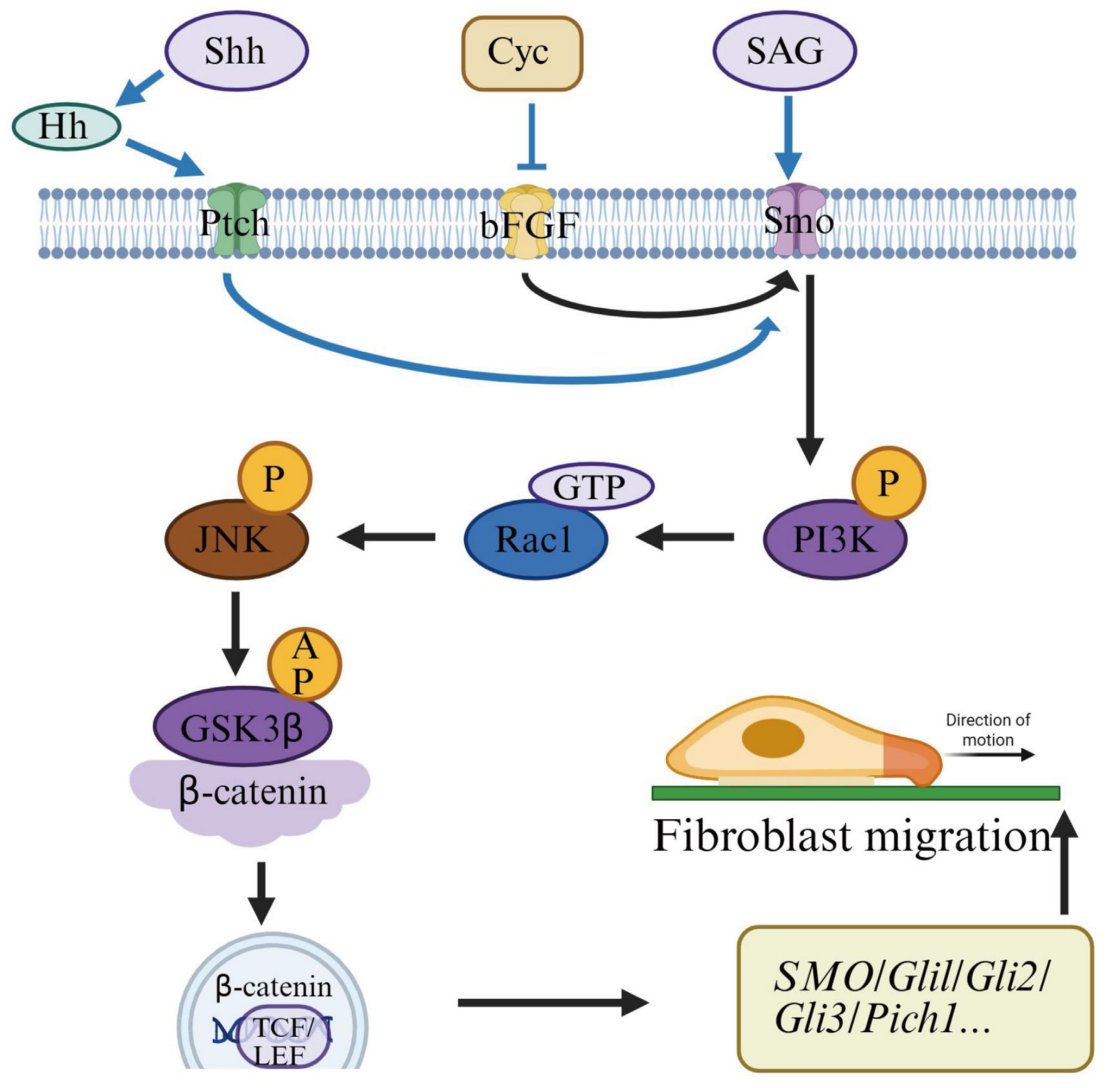
Schematic diagram of the migration process and mechanism of fibroblasts.

After myocardial necrosis or damage, the migration of cardiac fibroblasts to the infarcted area plays an important role in the repair process. The metabolites of early hypoxic cardiomyocytes can induce the migration of cardiac fibroblasts, while TNF-α and IL-1β can act as initial chemotactic inducers 81, particularly in response to the dynamic microenvironment following AMI. In the early stages of cardiac repair, fibroblasts undergo phenotypic transformation driven by inflammatory cues within the infarcted myocardium. Upon myocardial cell necrosis in the infarct region, a robust inflammatory response is triggered. Activated fibroblasts adopt a pro-inflammatory phenotype, initiating inflammasome activation and producing various cytokines and chemokines (Figure 3) 82. This pro-inflammatory behavior precedes their differentiation into myofibroblasts and persists until necrotic cells and matrix debris are completely cleared. Therefore, the resolution of inflammatory infiltration is closely associated with fibroblast migration and functional switching.

Fibroblasts are ubiquitously distributed and act as tissue-resident sentinel cells. During tissue injury, fibroblasts not only produce pro-inflammatory mediators but also facilitate leukocyte recruitment to damaged areas. One key mechanism involves the CD40 signaling pathway: CD40 expression on fibroblasts can be activated in an autocrine manner to synthesize chemokines, while also responding to infiltrating immune cells through CD40-CD40L (CD40 ligand) interactions. For example, CD40-mediated fibroblast activation leads to the secretion of interleukin-8 (IL-8), which recruits neutrophils and T lymphocytes to the injury site, promoting inflammation resolution and tissue repair 83. Fibroblasts also produce high levels of prostaglandin E2 (PGE2), which modulates the inflammatory microenvironment post-AMI. PGE2 can alter the phenotype of immature T lymphocytes by promoting Th2 differentiation and enhancing IL-5 synthesis, while suppressing IL-2 production in Th1 cells. This shift in the Th1/Th2 balance influences T cell migration and function 84. Consequently, fibroblasts serve as crucial immune modulators in AMI and shape a distinct immunological niche in PGE2-rich regions.

In addition to their immune functions, fibroblasts are key regulators of angiogenesis. They are an important source of VEGF, a potent pro-angiogenic factor also involved in tumor vascularization. VEGF expression by fibroblasts increases significantly during AMI, where it facilitates neovascularization and endothelial cell migration 85. Fibroblasts may store VEGF in an inactive state within the extracellular matrix, enabling rapid release and activation upon myocardial injury, thereby coordinating an emergency angiogenic response.

Furthermore, cardiac fibroblasts overexpress cardiac-specific transcription factors such as GATA-4 and GATA-6. *In vitro* co-culture studies have shown that endothelial cells exhibit impaired angiogenic responses when cultured with GATA4/6-deficient fibroblasts, confirming the regulatory role of fibroblasts in endothelial cell proliferation and migration 86. These findings highlight the active role of fibroblasts in orchestrating vascular formation and contributing to myocardial repair through paracrine and transcriptional regulation.

Given the central role of fibroblasts in inflammation regulation and angiogenesis, targeting their key signaling pathways holds significant therapeutic potential. Modulating the CD40-CD40L axis can appropriately suppress excessive inflammation, reduce leukocyte infiltration, and promote the orderly resolution of the immune response. Regulation of PGE2 synthesis or its EP2/EP4 receptor activity helps maintain the Th1/Th2 immune balance and optimizes the microenvironment for tissue repair. Additionally, enhancing VEGF expression and bioavailability through controlled-release systems or gene therapy can accelerate neovascularization, improve perfusion in the infarcted region, and facilitate myocardial regeneration. Taken together, fibroblasts are no longer viewed as passive matrix-producing cells but as active effectors in immune modulation and tissue remodeling. Their migration and phenotypic transformation after myocardial infarction serve as a critical link between inflammatory response and tissue repair, positioning them as promising targets for future therapeutic interventions.

#### Endothelial cell migration

Under normal circumstances, endothelial cells are quiescent, with little proliferative or migratory activity 87. However, when myocardial infarction occurs, the damaged tissue sends out signals that activate endothelial cells, prompting them to proliferate and migrate to form new vessels. In the infarcted area, vascular endothelial cells are impaired, and upon stimulation by basic fibroblast growth factor, the cells near the wound edge migrate directionally to the denuded area. The leader cells at the edge of the injury transmit migration signals to the follower cells. Leader cells form a thin layer by sensing chemical attractants, adhering to the basement membrane, while the following cells extend lamellipodia, which is mediated by VE-cadherin and α-catenin for migration 88. Without stimulation, the migration of endothelial cells is random.

Recent studies underscore the critical involvement of long noncoding RNAs (lncRNAs) in atherosclerosis by influencing the proliferation and migration of human umbilical vein endothelial cells. Deletion of the vascular homeostasis maintaining Tie1 and Tie2 receptor tyrosine kinases affects the proliferation and sprouting of cardiac endothelial cell. However, this effect does not affect the endothelial cells of the lungs and kidneys, without to neovessel formation in the heart 89, 90.

There are two important pathways that affect endothelial cell migration: the VEGF and Notch signaling pathways. Myocardial hypoxia in the infarcted area causes local tissue to secrete VEGFA, which binds to VEGFR2 on endothelial cells, regulating their proliferation and migration. Moreover, the VEGF signaling pathway can induce the expression of Notch ligands, such as Delta-like 4 (Dll4), which activates Notch signal transduction, inhibiting angiogenic sprouting 91. IIntegrins also play a crucial role in endothelial cell migration. Integrins are important mediators of endothelial cell migration, with various receptors related to angiogenesis, including collagen receptors (α1β1, α2β1), laminin receptors (α3β1, α6β1, α6β4), fibronectin receptors (α4β1, α5β1), and αv receptors (αvβ3, αvβ5) 92, 93. For example, αvβ3 regulates the directional migration of endothelial cells 94. VEGF-induced cell migration requires the mediation of αvβ3, αvβ5, and β1 integrins 95. Studies have been conducted to promote angiogenesis after AMI by increasing the production of VEGFA 96. Furthermore, ablation of serum- and glucocorticoid-induced kinase 1 (SGK1) can reduce the phosphorylation of its target protein NDRG1, affecting endothelial cell migration and causing tubular formation defects, thereby reducing neovascularization 97.

#### Stem cell migration

**Figure 4:**
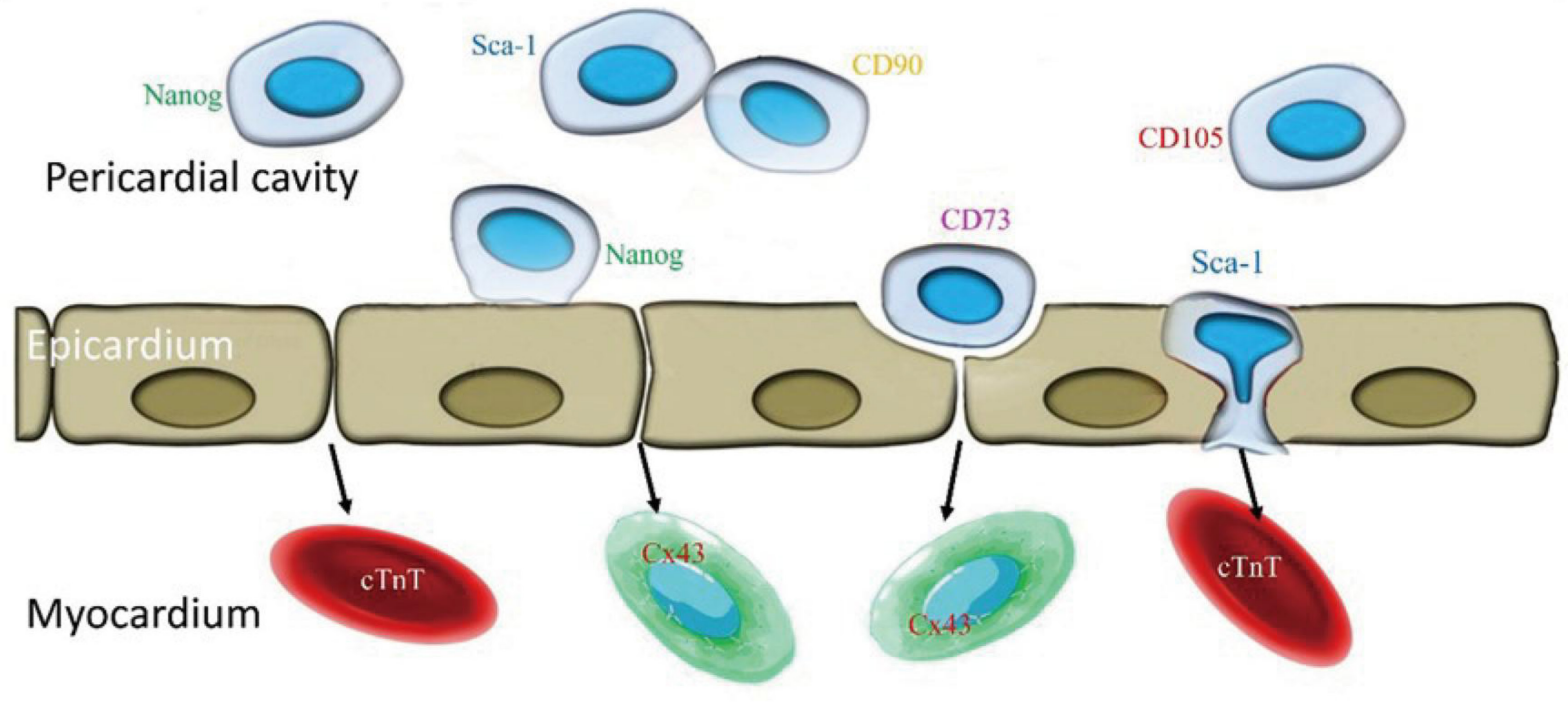
Schematic diagram of migration of stem cells in pericardial fluid to myocardial tissue.

After a MI, the injury microenvironment of cardiac tissue undergoes significant changes, including hypoxia, an inflammatory response, and the release of chemokines. These factors collectively guide stem cells to migrate to the infarcted area and exert their repair functions. Bone marrow MSCs, cardiac progenitor cells (CPCs), cardiac stem cell antigen Sca-1 98, 99, Nanog-positive cells 52 and human pericardial fluid stem cells (hPFCs) 100 can migrate to the ischemic myocardium in response to chemokine signals (such as SDF-1/CXCR4 and the HGF/c-Met axis). Our research found that stem cells in pericardial fluid can freely migrate from the epicardium to myocardial tissue, whether it is a normal heart or a diseased heart (Figure 4) 100. However, this migration process is often limited by a variety of factors, including insufficient cell migration ability, transient expression or insufficient concentration of chemokines, and the complexity of the host's damaged tissue microenvironment. These unfavorable factors can be addressed by enhancing migration ability through gene editing, chemokine signal enhancement, or biomaterials (such as hydrogels). In addition, cell adhesion factors (such as ICAM-1/VLA-4 and LFA-1) can promote stem cells to adhere to and penetrate the vascular wall, allowing them to enter the injured tissue. The hypoxic microenvironment can upregulate the expression of chemokine SDF-1 through HIF-1α, enhancing the homing ability of stem cells, while inflammatory factors such as IL-6 and TNF-α secreted by immune cells (such as macrophages and T cells) can also affect stem cell migration and survival. Therefore, optimizing stem cell migration strategies, such as improving responsiveness to chemokines or combining biomaterials to enhance microenvironment adaptability, may help improve the efficacy of stem cell therapy for myocardial infarction.

Many chemokines are upregulated in the infarcted heart, suggesting that they play a role in regulating the inflammatory response 101. After myocardial ischemia, released chemokines include CCL2 (MCP-1), CCL3 (MIP-1), CCL4 (MIP-1β), CXCL8 (IL-8), CXCL10 (IP-10), and CXCL12 (SDF-1) 102. Myocardial ischemia is accompanied by cellular hypoxia, which can significantly promote cell migration 103. Hypoxia affects the microenvironment by inducing the expression of hypoxia-inducible factor-1 alpha (HIF-1α), upregulating the production of chemokines such as SDF-1, and enhancing chemotaxis toward stem cells. During the inflammatory response, immune cells such as macrophages and T lymphocytes secrete large amounts of inflammatory factors like IL-6, TNF-α, and IL-1β. These factors can promote the migration of stem cells to the damaged area while also potentially having negative effects on the survival and function of these stem cells, thereby limiting the effectiveness of stem cell therapy.

**Figure 5:**
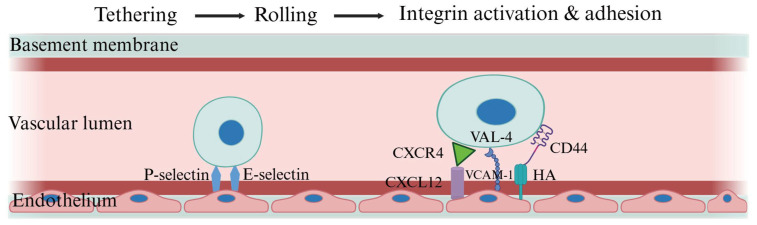
Schematic diagram of stem cell migration along vascular endothelial cells. One of the key steps for stem cells to enter the infarcted area is to adhere to and penetrate the vascular endothelial cell barrier. Cell adhesion factors such as intercellular adhesion molecule-1 (ICAM-1) and its receptors VLA-4 and LFA-1 play a crucial role in the endothelial adhesion and transendothelial migration of stem cells. Optimizing the expression or function of these adhesion factors can enhance the efficiency of stem cell homing.

In addition, after the capillary endothelium is activated, E-, I-, and P-selectin (cell adhesion molecules) are rapidly presented on the endothelial surface, triggering leukocyte rolling. Chemokines released during inflammation induce the adhesion and chemotaxis of leukocytes, and the type of chemokines released can determine the subtype of migrating leukocytes. Integrins are usually in a low-affinity state but can be switched to a high-affinity state by the action of chemokines. Firm adhesion penetrates between the endothelial tight junctions and the basement membrane, allowing access to the ECM of the local tissue matrix. Here, cells adhere to ECM components such as hyaluronic acid, laminin, collagen, and fibronectin through integrins, CD44, and other cell adhesion molecules. Migration through the ECM is facilitated by ECM-degrading enzymes like matrix metalloproteinases (MMPs), allowing cells to move along gradients of chemotactic factors within local tissues (Figure 5) 104, 105.

Currently, strategies to enhance stem cell migration and homing capabilities include: (1) gene editing techniques, such as CRISPR/Cas9-mediated genetic modifications, to increase the expression levels of chemokine receptors on the surface of stem cells, like CXCR4; (2) enhancement of chemokine signaling pathways, for example, by using exogenous chemokines or slow-release carriers to elevate local chemokine concentrations; (3) bio-material assistance, such as employing hydrogel carriers with good biocompatibility and controlled release capabilities to continuously release chemokines and provide a favorable microenvironment for stem cells, thereby significantly enhancing their directed migration towards damaged areas^11^, ^106^.

In summary, by comprehensively applying gene editing, chemokine signaling enhancement, and biomaterial-assisted techniques, it is expected that the migration efficiency, homing ability, and survival rate of stem cells in the damaged myocardial region will be significantly improved, thereby enhancing the clinical efficacy of myocardial infarction treatment. The optimization of these strategies holds significant research value and application potential for the further clinical translation of stem cell therapy for myocardial infarction.

#### Dendritic cells migration

DCs are a crucial component of the body's immune system. They uniquely regulate both acute and chronic inflammatory responses. Both DCs and monocytes are key inflammatory cells and can play a role in cardiac reverse remodeling following cardiac resynchronization therapy 107, 108. The immune response to myocardial pathological injury caused by different etiologies requires highly coordinated interactions between cardiac cells and immune cells. Cardiac fibroblasts are the dominant cell type in the heart. Their functional effects include expressing contractile proteins and exhibiting increased migration, proliferation, and secretion characteristics. Cardiac fibroblasts, dendritic cells, macrophages, CD4+ T cells, and other immune cells are known to play different roles in the pathogenesis of myocarditis. Cardiac fibroblasts are not passive players in the immune response, as evidenced by their release of soluble signals and/or direct interactions with immune cells. Generally, fibroblasts participate in the synthesis of cytokines, chemokines, prostaglandins, matrix components, and matrix-degrading enzymes, which affect the function of dendritic cells, CD4+ T cells, and macrophages in myocarditis, and vice versa. This further confirms the existence of a dialogue between cardiac fibroblasts and immune cells recruited into the myocardial microenvironment during myocarditis 109.

DCs migrate from peripheral blood to the infarcted area after MI, a process regulated by CCR2, CCL2, and GM-CSF signals. In myocardial tissue, necrotic cardiomyocytes release danger signals, and inflammatory cells such as dendritic cells, neutrophils, monocytes, and macrophages migrate to the site of cardiac injury to clear cellular debris and secrete various inflammatory factors to activate the inflammatory response 110. The immune process of myocardial pathological injury caused by different etiologies requires cardiac cells and immune DCs to take up antigens in the infarcted area and further migrate to peripheral lymph nodes to activate T cells, thereby regulating inflammation and the immune response. DCs mainly include cdc1, cdc2, and pDCs, which have different roles in inflammation resolution, fibrosis, and cardiac repair. These subpopulations can be distinguished by differences in surface markers in multiple tissues, such as XCR1+ CADM1+ CD172a- CDC1s and XCR1- CADM1- CD172a+ CDC2s 111. Additional tissue-specific markers, such as spleen CD8α+ CDC1 and CD4+ CDC2 or lung CD103+ CDC1 and CD11b+ CDC2, can also be used for distinction. It should be noted that DC subpopulations exhibit significant plasticity, depending on their microenvironment. Different cell subtypes have different immune effects. For example, pro-inflammatory cdc1 mainly activates the Th1 immune response, while pDCs participate in immune regulation by secreting IFN-α and may play a role in immune tolerance and inflammation suppression.

Additionally, the migration of DCs is influenced by chemokines (such as the CCR2-CCL2 axis) and damage-related molecular patterns (DAMPs, such as HMGB1 and HSPs). These signals activate DCs and enhance their migration ability. Due to the key role of DCs in the myocardial inflammatory response, research on how to accurately regulate their migration and function will help optimize immune intervention strategies after MI 112, 113.

#### Pericytes migration

Pericytes play a key role in stabilizing the microcirculation after vascular remodeling 114, 115. The role of pericytes in the remodeling process caused by myocardial ischemia remains unclear. Quijada et al. used multiple lineage tracing mouse models and found that pericytes actively migrate to the site of myocardial injury and express fibrotic genes, which aligns with the increased leakage of vascular contents after MI. This result suggests that cardiac pericytes play a crucial role in controlling vascular homeostasis and the fibrotic response following acute ischemic injury. This information will help guide new strategies to maintain vascular integrity and mitigate pathological cardiac remodeling 116.

Pericyte migration primarily occurs during active angiogenesis and vascular remodeling. Pericytes migrate to the site of injury after myocardial infarction and, in collaboration with endothelial cells, promote the formation of new blood vessels. Their migration is regulated by the PDGF-BB/PDGFR-β axis, while TGF-β1 can influence their phenotypic transition 117, 118, 119-121.

Pericytes must dissolve their own and the specialized ECM surrounding endothelial cells before detaching from the capillary wall, commonly known as the vascular basement membrane. It is known that pericytes secrete MMPs, such as MMP2, MMP3, and MMP9, during physiological and certain pathological angiogenesis processes, which help pericytes detach from the capillary wall 122, 123. Key mechanisms of pericyte migration involve regulation by chemokines and growth factors, such as PDGF-BB, which promotes pericyte recruitment around newly formed capillaries and stabilizes vascular structures, while VEGF-A enhances pericyte migration towards vascular sites and promotes angiogenesis. Additionally, TGF-β1 influences pericyte phenotypic transition, partially differentiating them into myofibroblasts, thereby promoting cardiac fibrosis. ECM remodeling is also crucial during pericyte migration; for instance, MMPs degrade ECM to facilitate pericyte migration, and integrins mediate interactions between pericytes and the matrix, affecting their adhesion and motility.

Furthermore, the inflammatory microenvironment plays a critical role in regulating pericyte migration and differentiation. Pro-inflammatory signals (such as TNF-α, IL-1β) promote the transdifferentiation of pericytes into myofibroblasts, exacerbating fibrosis, whereas anti-inflammatory factors (like IL-10) may inhibit this process, reducing fibrosis occurrence. Therefore, pericytes have dual roles post-MI: on one hand, they secrete angiogenic factors to promote microvascular formation, aiding in restoring cardiac blood supply; on the other hand, their transdifferentiation into myofibroblasts may lead to increased collagen deposition, increasing cardiac stiffness. Thus, future research should focus on precisely regulating pericyte fate to promote angiogenesis while minimizing fibrosis 124.

Overall, the migration of different cell types after MI is regulated by chemokines, inflammatory signals, and cell-to-cell interactions, collectively determining the final outcome of cardiac repair. However, several key issues remain unresolved, such as how to precisely control the migration of stem cells and immune cells to enhance myocardial repair capacity. How do the roles of T cells and DCs change during the early and late stages of MI? Can different subtypes of immune cells be targeted for regulation? How can the fate of pericytes be modulated to promote angiogenesis while reducing cardiac fibrosis? Future research can combine single-cell sequencing, spatial transcriptome analysis, and live imaging techniques to further explore the dynamic changes in cell migration after MI and develop more precise cell therapy strategies.

In short, cell migration after AMI is a complex process that is essential for tissue regeneration and wound healing. Although the mechanism of some cell migration is still unclear, anti-inflammatory, fibrotic, and angiogenic drugs can be developed to target the signals involved in myocardial infarction cell migration. These drugs can block pathologically related migration without interfering with physiological cell functions.

### Heart failure and cell migration

Although the causes of heart failure vary, they all share a common feature: structural and functional remodeling. There are significant differences in cell migration patterns between AMI and heart failure (HF) (Table 2). In AMI, immune cells are primarily involved in the clearance of necrotic tissue and initial repair in the early stages after injury, with a focus on repairing ischemic damage. During this process, hypoxia and fibrosis are triggered by the loss of capillaries and the adverse remodeling of ventricular arterioles 125. Fibrosis occurs in almost every form of heart disease, but before fibrosis develops, angiogenesis is an early attempt by the injured heart to compensate for the increased oxygen demand of hypertrophic cardiomyocytes. This later results in capillary loss, cell death, and replacement fibrosis 126.

In HF, hypertrophic remodeling often occurs in the heart wall, involving fibroblasts, endothelial cells, immune cells, vascular endothelial cells, and smooth muscle cells 127. HF is a chronic process with a more complex migration mechanism that is both protective and potentially pathogenic. Migrating cells gradually shift from early repair to pathological fibrosis and sclerosis, promoting the progression of adverse remodeling. Therefore, although AMI and HF share similar immune responses in the initial stages, in the chronic stage, the role of immune cells gradually shifts to exacerbate the pathological process, eventually leading to the deterioration of HF.

In conclusion, the cell migration mechanism in AMI aims at “rapid clearance + local repair”, with an intense and transient signal response. In HF, a chronic process, the migrated cells are both protective and potentially pathogenic. Immune cell migration is prolonged, and subpopulations change more significantly. The migration mechanism of the same cell type differs in the two conditions: for example, TREG migration is reparative in AMI but may transform into Th17 in HF, contributing to fibrosis 128. During HF, the migration and functional changes of various cell types collectively influence cardiac remodeling and disease progression.

#### Neutrophil migration

During the development of HF, changes in the cardiac microenvironment are influenced and controlled by various immune cells, such as macrophages, neutrophils, dendritic cells, eosinophils, and T lymphocytes, as well as the cytokines they produce 128. Among these, neutrophils continue to migrate to the injury site, regulated by CXCL1, CXCL8 and GM-CSF signals. Although they can phagocytose necrotic cells and clear damaged tissues, their prolonged presence may lead to a sustained inflammatory response, increase oxidative stress, and promote the release of cytokines (such as TNF-α and IL-1β), exacerbating myocardial injury and fibrosis. The CC chemokine CCL2 exerts fibrotic effects by recruiting and activating monocytes and macrophages through its receptor CCR2. CXC chemokines containing ELR motifs can exert profibrotic effects by recruiting activated neutrophils, leading to the formation of neutrophil extracellular traps (NETs), or by activating fibrogenic monocytes. CXCL12 also exerts fibrotic effects through its impact on fibroblasts and immune cells. In contrast, the CXCR3 ligand CXCL10 can reduce cardiac fibrosis and inhibit fibroblast migration. Chemokines may be promising therapeutic targets for patients with HF accompanied by significant inflammation and fibrosis 129. Furthermore, neutrophils release MMPs, which may exacerbate the degradation of the ECM, leading to instability in cardiac remodeling. Therefore, the abnormal recruitment and persistent activation of neutrophils could be key factors in the progression of HF.

#### Monocyte and macrophage migration

During the progression of HF, monocytes are continuously recruited from the circulatory system to the heart, where they further differentiate into M1 or M2 macrophages. This recruited process is regulated by the CCL2-CCR2 signaling axis. Recently, it has been discovered that CCL17 may also act as an inflammatory mediator for CCR2+ macrophages and dendritic cells. Inhibiting CCL17 could potentially be an effective approach to promote TREG recruitment and suppress myocardial inflammation 130. Ang II-activated macrophages play a key role in subcellular defects and adverse cardiac remodeling during HF progression. Ang II stimulates macrophages through its AT1 receptor, leading to the release of oxygen free radicals, cytokines, chemokines, and other inflammatory mediators in the myocardium. It also upregulates the expression of integrin adhesion molecules on monocytes and endothelial cells, facilitating interactions between monocytes and endothelial cells. The transendothelial migration of monocyte-derived macrophages has significant biological effects, including fibroblast proliferation, ECM protein deposition, the induction of intravascular/interstitial fibrosis, cardiac hypertrophy, and the progression of HF 131.

M1 and M2 macrophages play different roles in the process of HF. M1 macrophages primarily secrete proinflammatory cytokines (such as TNF-α, IL-6, IL-1β), which may aggravate chronic inflammation and myocardial injury, while M2 macrophages promote repair and fibrosis by secreting IL-10 and TGF-β. However, in the environment of chronic HF, the prolonged presence of macrophages may lead to excessive ECM deposition, which exacerbates cardiac fibrosis and stiffness. Therefore, balancing the ratio of M1/M2 macrophages may be an important strategy to regulate the progression of HF.

#### Fibroblast migration

Activated fibroblasts aid in disease progression such as Duchenne muscular dystrophy, hypertrophic cardiomyopathy, and dilated cardiomyopathy 132. Cardiac fibrosis is a late stage pathological feature of various cardiovascular diseases, and important functional changes include abnormal activation and migration of cardiac fibroblasts, as well as excessive and disordered deposition of extracellular matrix 133. Pathological stimulation is one of the important factors in fibroblast migration.

During the fibrosis process in HF, fibroblasts continuously migrate to the myocardial injury site, primarily regulated by TGF-β, PDGF, and CTGF (connective tissue growth factor). Cardiac fibroblasts can produce pro-inflammatory mediators and act as sentinel cells activated by mechanical stress. These cells are capable of recruiting inflammatory cells to cardiac tissue, a process known to worsen patient outcomes 134. Hypoxia-induced mitogenic factor (HIMF) induces cardiac fibrosis through paracrine effects on cardiomyocytes. IL-6, a downstream signal of HIMF, plays a central role in cardiomyocyte hypertrophy and cardiac fibrosis by mediating the activation of the MAPK and CAMKII-STAT3 pathways 135. Following MI, the expression of Inter-α trypsin inhibitor heavy chain 5 (ITIH5) is upregulated, accelerating ECM-fibroblast-macrophage interactions. This promotes macrophage phenotypic transformation, CFS activation, and cardiac fibrosis remodeling, ultimately leading to HF 136.

Since the occurrence and progression of HF is a prolonged pathological process, fibroblasts are continuously activated by the aforementioned factors, ultimately leading to excessive ECM deposition and cardiac fibrosis. This increases myocardial stiffness and reduces cardiac diastolic function. Additionally, some fibroblasts can transdifferentiate into myofibroblasts, further enhancing ECM synthesis capacity and exacerbating myocardial remodeling. Therefore, limiting the abnormal migration and activation of fibroblasts may be an important strategy to reduce the progression of HF.

#### Endothelial cell migration

The proliferation and migration of endothelial cells are critical to angiogenesis within the myocardium. Endothelial-to-mesenchymal transition (EndMT) is also a form of endothelial cell migration, during which endothelial cells detach from the endothelium, migrate and lose their endothelial characteristics while acquiring mesenchymal features. Subsequently, endothelial cells lose lumen-channel polarity, extend spindle-shaped filaments, and migrate outside the vascular wall, losing their endothelial properties 137. Autocrine VEGF-B signaling from endothelial cells does not promote VEGF-B-induced endothelial cell migration or contribute to myocardial capillaries but can lead to pathological cardiac hypertrophy 138. During HF, endothelial cells maintain their migratory ability and contribute to angiogenesis. VEGF-A, angiopoietin-1 (Ang-1), and the Notch signaling pathway regulate the migration of endothelial cells to ischemic areas and promote microvascular formation to sustain myocardial perfusion. However, during the development of HF, angiogenic capacity is often inhibited, leading to capillary rarefaction, which exacerbates myocardial ischemia and hypoxia, further deteriorating cardiac function. Additionally, endothelial dysfunction may result in the release of inflammatory factors and disruption of the endothelial barrier, affecting the cardiac microenvironment. Therefore, enhancing endothelial cell migration and promoting vascular reconstruction may help improve cardiac function in HF patients.

#### MSCs migration

Cardiac stem cell migration includes both resident stem cell migration and foreign stem cell migration. The former refers to the movement of stem cells within the heart muscle, while the latter refers to the arrival of stem cells in the heart via blood circulation or injection. We previously injected pericardial fluid-derived stem cells and adipose-derived mesenchymal stem cells into the pericardial cavity of rats. These transplanted stem cells were able to penetrate the epicardium, enter the myocardium, and migrate freely within myocardial tissue to play a therapeutic role in HF. Their therapeutic effects are achieved through anti-inflammatory, anti-fibrotic, and angiogenesis-promoting mechanisms 100, 139, 140. Intracardiac injection of human umbilical cord-derived mesenchymal stem cells (HucMSCs) aids in cardiac function recovery and reduces cardiac remodeling after myocardial infarction, while also promoting the migration of CD4+ T cells to the injured heart 141. During the development of HF, the SDF-1/CXCR4 axis is an important signaling pathway regulating the homing of MSCs to the heart, while TGF-β and IGF-1 can also promote the migration and differentiation of MSCs. In HF models, MSCs secrete growth factors (such as VEGF and HGF) through paracrine signaling, promoting angiogenesis, anti-inflammation, and anti-fibrosis 142. Additionally, MSCs can regulate the inflammatory response and reduce myocardial injury by interacting with immune cells (such as macrophages and T cells). However, in the chronic inflammatory environment of HF, the survival rate and migration ability of MSCs may be reduced. Therefore, strategies to optimize the migration ability of MSCs (such as gene modification or biomaterial assistance) may help enhance their therapeutic effects.

#### T cell migration

During HF, T cells continuously migrate to cardiac tissue, where CD8+ T cells may exacerbate myocardial damage, while regulatory T cells (Tregs) may have a protective effect. CCL5 and the CXCL9/CXCL10-CXCR3 axis promote T cell recruitment in HF, and IFN-γ and IL-17 can further enhance T cell infiltration. CCL21 is an effective regulator of T cell migration to non-lymphoid tissues, potentially exhibiting inflammatory properties and influencing tissue remodeling 143. MyD88 (Myoid differentiation response 88) regulates T cell activation and survival through TCR-dependent rather than TLR-dependent signaling. T cell activation leads to cardiac fibroblast transformation and maladaptive cardiac remodeling. The intrinsic role of MyD88 in limiting T cell activation is central to the regulation of cardiac inflammation during cardiac adaptation to stress 144.

During chronic HF, T cells may maintain a state of low-grade inflammation, leading to persistent myocardial injury and fibrosis progression. Tregs suppress inflammation by secreting IL-10 and TGF-β, reduce fibrosis, and may improve cardiac function. Therefore, targeting the regulation of T cell migration and function could be a potential strategy for immune intervention in HF.

#### DC migration

During the HF process, DCs migrate through the bloodstream to the myocardial tissue. The mechanism for DC migration is as follows: Immature DCs patrol their environment by engulfing extracellular material. DC migration and antigen capture are antagonistic, caused by the transient enrichment of myosin IIA at the cell front, which disrupts the back-to-front gradient of motor proteins, slowing movement but enhancing antigen capture. The enrichment of myosin IIA at the cell front requires the invariant chain (II) associated with MHC II. Therefore, by controlling the localization of myosin IIA, it imposes an intermittent antigen capture behavior on dendritic cells, potentially promoting environmental patrolling 145.

DCs activate T cells through antigen presentation in myocardial tissue and regulate the inflammatory response. Regulated by CCL2, CCR7, and GM-CSF signaling pathways, DCs may maintain low-level inflammation during chronic HF and affect the progression of cardiac fibrosis. The precise regulation of DC migration and function is of great significance for immunotherapy in HF.

#### Pericyte migration

During the development of HF, the PDGF-BB/PDGFR-β signaling pathway is a crucial regulator of pericyte migration, while TGF-β1 may promote the transdifferentiation of pericytes into myofibroblasts, enhancing their ECM synthesis capacity and exacerbating myocardial fibrosis. Additionally, in chronic HF, the migratory capacity of pericytes may decrease, leading to the loss of microvessels and insufficient myocardial perfusion. Therefore, modulating pericyte migration and balancing their pro-angiogenic and anti-fibrotic effects may have significant implications for improving HF prognosis.

In summary, during the progression of HF, the migration and functional changes of different cell types jointly affect cardiac remodeling and disease progression. Among these, the abnormal recruitment of neutrophils, T cells, and M1 macrophages may exacerbate the inflammatory response, while M2 macrophages, MSCs, and regulatory T cells (Tregs) may promote repair. Additionally, the migration of fibroblasts, pericytes, and endothelial cells plays a crucial role in regulating myocardial remodeling and maintaining vascular homeostasis. Cytokines and chemokines act as key regulators of cell migration in these processes, directly affecting the severity of the disease. In the treatment of cardiovascular diseases, blocking specific and non-specific cytokines and chemokines can inhibit the migration of inflammatory and immune cells, thereby reducing the inflammatory and immune responses at the site of myocardial injury. For example, the administration of small molecules that inhibit cytokines and chemokines can alleviate inflammation, while the local release of chemoattractants can prevent cells from migrating to the wound site. Therefore, future research should focus on elucidating the cell migration pathways related to HF, with an emphasis on accurately regulating cell migration, reducing harmful inflammation, and improving repair mechanisms to enhance the clinical therapeutic effects for HF.

## Spaceomics technology breaks through the limitations of research techniques for “dynamic cell migration *in vivo*”

Traditional *in vivo* cell migration research has long relied on methods such as fluorescence tracing, live cell imaging, flow cytometry, and single-cell sequencing, but there are three inherent limitations: 1. Lost spatial coordinates: Single cell sequencing dissociates tissues, making it impossible to know where cells come from and go. 2. Resolution and flux cannot be achieved simultaneously: Imaging can show location, but it is difficult to simultaneously obtain whole genome expression information. 3. Unable to analyze the driving mechanism of microenvironment: Migration is not only the behavior of cells themselves, but also regulated by surrounding cells, matrix, and signal gradients, but traditional techniques are difficult to quantify *in situ*. Spatial transcriptomics, spatial proteomics, spatial metabolomics and other spatial omics technologies break through the limitations of these three levels from the following aspects 146-150.

### Accurately locate the characteristics of *in situ* migrating cells in tissues

Traditional single-cell sequencing can identify subpopulations of migrating cells (such as EMT, highly migratory stem cells, and invasive frontier cells), but their precise location within the tissue is unknown. Space omics enables unbiased, panoramic transcriptome/protein detection of tissues *in situ*. Directly map the expression profiles of migration related genes to tissue anatomical structures. This technology clearly distinguishes the starting area, migration path, and destination of cell migration, achieving simultaneous analysis of “cell identity + spatial position + expression status”.

### Reconstruction of cellular dynamic trajectories in tissue space cells

In the past, cell trajectories relied on pseudotime analysis, but only calculated trajectories and lacked physical spatial validation. Space omics constructs a 3D spatial structure through continuous slicing of spatial maps, projects pseudo temporal sequences onto spatial coordinates, and combines time series samples (different stages of development/injury/tumor) to reconstruct cell migration paths in real tissue space, achieving the transition from “virtual trajectory” to “physical spatial trajectory” and from “static state” to “dynamic migration process”. This is a spatiotemporal joint inference that traditional technology cannot achieve at all.

### Micro signal regulation of cell directed migration and interaction with neighboring cells

Cell migration is not a random movement, but is guided by chemokines, matrix stiffness, and signals from neighboring cells. Space omics can identify the spatial neighbors of migrating cells *in situ*, analyze the spatial co-localization of ligand receptor pairs, plot the spatial distribution of chemokine gradients and signaling pathways, and reveal the *in situ* interaction network between “guiding cells” and “migrating cells.”

### Promoting cell migration research from phenotype observation to mechanism exploration

Traditional imaging can only observe a few markers, while spatial genomics provides a whole transcriptome/whole proteome dimension to systematically screen key genes/pathways driving migration, discover unknown migration subgroups and new migration phenotypes, and achieve mechanism level discovery in tumor invasion, development, immune homing, and damage repair, upgrading cell migration research from phenotype observation to molecular mechanism analysis. From “looking at a few proteins” to “panoramic molecular mechanisms”.

### Deep exploration of molecular mechanisms for achieving panoramic cell migration

The new generation of spatial genomics, such as MERFISH, seqFISH, DBiT seq, and spatial proteomics, is achieving subcellular resolution, co-localization of multiple proteins and transcripts, and linkage with *in vivo* imaging for long-term and dynamic tracking of migration events. We are moving towards high spatiotemporal resolution, *in vivo*, and dynamic direction, ultimately achieving the ability to see where cells are, where they migrate, what they express, and who regulates them in intact tissues.

In summary, spatial omics technology fundamentally breaks through the three bottlenecks of position loss, insufficient flux, and ambiguous microenvironment mechanisms in traditional cell migration research by preserving spatial position, coupling single-cell resolution, panoramic molecular mapping, and *in situ* interaction analysis. It shifts the tracking of dynamic cell migration *in vivo* from “indirect inference” to “*in situ* panoramic analysis”, providing unprecedented research paradigms for fields such as development, immunity, tumor metastasis, and tissue regeneration 151-154.

## Challenges and future directions in cell migration

Cell migration plays a vital role in the onset, progression, and repair of cardiovascular diseases. By precisely regulating cell migration, it is possible not only to effectively promote cardiac tissue regeneration and repair, but also to alleviate excessive inflammatory responses and pathological fibrosis. Although many academic papers on basic research of cell migration have been published, they have not received much attention in clinical applications. Therefore, targeted regulation based on the migratory characteristics of different cell types is becoming a new therapeutic direction for cardiovascular diseases. However, achieving efficient and precise control of cell migration still faces multiple challenges (Table 3).

### Existing issues

#### Insufficient understanding of microenvironment complexity

Current research on cell migration primarily involves single-cell studies *in vitro* or tissues/organoids of lower animals, which struggle to replicate the true scenario of human organs. Cells within the body are interconnected and exist in a collective form; the extracellular matrix is a complex system with viscoelasticity, dynamic stiffness, stress relaxation, and uneven fiber alignment. Even within the same tissue, the microenvironment exhibits heterogeneity. The microenvironments in tumors, inflammatory sites, and tissue repair areas are highly heterogeneous, leading to significant differences in migration behaviors across different regions of the same tissue. Traditional 2D rigid planes and 3D static collagen models fail to simulate these conditions, resulting in completely distinct migration modes (mesenchymal/deformable/parachute) and velocities between *in vivo* and *in vitro* settings 155. Whether many findings can be validated in humans remains unknown, thus limiting the clinical relevance of migration theories.

#### The mechanism of cell migration is not yet fully understood

Cell migration is the result of the synergy of actin cytoskeleton, adhesive plaques, contractility, matrix remodeling, and signaling pathways (Rho GTPase, PI3K, calcium signaling), but the integration mechanism of molecular mechanics signaling pathways is unclear. The cross-scale coupling mechanism (molecular → subcellular → cells → tissue) is still incomplete 156. Some diseases, such as tumors, often suffer from issues of cell super diffusion/sub diffusion (non-Brownian motion) and stress relaxation in viscoelastic matrices. Research on how to regulate abnormal diffusion mechanisms is just beginning. Basic research must be strengthened until the fundamental theories are elucidated in order to approach clinical applications more closely 155, 157.

#### *In vivo* transformation and clinical application

There are many barriers between cell migration from basic research to clinical application. Difficulty in tracking and manipulating the body is a major bottleneck. The currently used live imaging has low resolution, weak signal, and difficulty in long-term tracking. There is a lack of safe and efficient methods for precise manipulation of specific cell migration, such as enhancing T cell infiltration and inhibiting cancer cell metastasis. After delivering seed cells through cell transplantation, the migration targets of cells (such as Rho and focal adhesion kinase) are mostly broad-spectrum pathways with significant side effects. Currently, the lack of specific targets (such as tumor microenvironment specific adhesion/degradation) is also a major issue 158.

There is significant heterogeneity in cell migration, and the migration ability of different cells varies greatly 159. Some immune cells exhibit low migration efficiency, which hinders disease recovery, such as T cells' poor infiltration in dense tumor stroma. However, cardiomyocytes are tightly connected by intercalated discs, making isolated cells rare. Through self-proliferation, the progeny of cardiomyocytes immediately form intercalated discs with adjacent cells, thus preventing significant positional movement. Consequently, relying on cardiomyocyte migration for therapeutic effects is highly limited in clinical practice 160.

In addition to the above, the current experimental techniques still have obvious shortcomings: for example, the most commonly used cell scratch experiment has large manual errors, is only 2D, and cannot distinguish between migration and proliferation. Although there are some reports on using 3D matrix to study cell migration, the operation is complex, imaging penetration is shallow, quantification is difficult, and the cost is high 161, 162. In addition, data-driven modeling, AI inference of cell migration mechanisms, physical models from molecular to tissue scales (such as active networks), organoids, and so on, still lack universality in practical applications and have a significant gap with *in vivo* reality 163-165. The solutions to these problems must be gradually achieved with the continuous advancement of technology.

### Future directions

#### In-depth investigation of migration mechanisms

A deeper understanding of the signaling pathways involved in cell migration is fundamental. For instance, the SDF-1/CXCR4 axis has been shown to significantly promote the directed migration of stem cells to injured cardiac regions 166. In the future, gene editing or small molecule drugs may be employed to precisely regulate these key pathways to enhance therapeutic efficacy.

#### Development of targeted therapies for cell migration

For example, enhancing CXCR4 expression in MSCs via genetic engineering can improve their responsiveness to SDF-1 gradients, significantly boosting their migratory efficiency 167. Based on this principle, targeted vectors or signaling modulators can be developed to achieve precise regulation of cell migration.

#### Optimization of scaffold and biomaterial design

The integration of nanotechnology offers new perspectives for studying cell migration. For example, nanofiber scaffolds not only provide physical support but also enhance cell adhesion and guided migration through surface modification 168. Shen et al. designed a nano-coated left atrial appendage occluder, which showed excellent rapid sealing and long-term safety in both animal experiments and clinical cases 169. The device promotes rapid endothelial cell migration, achieving complete endothelialization without prolonged medication, significantly reducing the risk of stroke and complications in atrial fibrillation patients. This study highlights the great potential of surface modification and structural design of biomaterials in promoting cell migration.

#### Enhancement of cellular biological properties

Studies have found that hypoxic preconditioning can improve MSC migration and anti-inflammatory abilities, enhancing their adaptability and functionality in cardiac tissue 170. Similar “cell activation” strategies may become key steps in improving the therapeutic effects of stem cell-based therapies in the future.

#### Multidisciplinary collaboration

The complexity of cardiovascular regenerative medicine necessitates multidisciplinary collaboration. Future research should integrate biology, materials science, pharmaceutical engineering, and biomanufacturing. For example, using 3D printing technology to construct scaffolds that mimic the cardiac microenvironment can provide precise support for cell migration and enhance regenerative outcomes 171.

## Figures and Tables

**Table 1:** A brief summary of the migration of different cell types in myocardial infarction.

Cell Type	Migration Timing	Regulatory Mechanisms	Functions	References
Cardiomyocytes	During embryonic development and after injury, unclear in healthy adult hearts	Mitochondrial metabolic suppression, IL-6, glucose metabolism, BMSC-derived IGF-1 enhances migration	Structural remodeling, renewal of cardiomyocytes, microstructural regulation	42-45
Neutrophils	Rapid migration within hours post-AMI; dramatic increase in number	Chemokines (CXCL1/2/5/8, CCL2/3/5), GM-CSF, Dectin-1, GDF-15; CD40-CD40L, IL-8	Debris clearance, recruitment of reparative cells; excessive migration causes ROS release and myocardial damage	46-53
Monocytes / Macrophages	Migrate within 30 min post-MI, replace resident macrophages within 1 day	CCR2/CCL2 axis, Ang II, IL-1α/β, CCL5, CCR9, Lgmn; B cell paracrine signals induce MHC-II	M1: pro-inflammatory and debris clearance; M2: anti-inflammatory and reparative; regulate remodeling and scar formation	54-65
Fibroblasts	Migrate to infarcted area in response to hypoxia	TNF-α, IL-1β, CD40-CD40L, PGE2, GATA4/6, VEGF; inflammatory signals induce myofibroblast transformation	Immune modulation, inflammation resolution, scar formation, angiogenesis; recruit neutrophils, regulate T cells	66-72
Endothelial Cells	Quiescent under homeostasis; activated and migrated post-AMI	VEGF-VEGFR2, Notch-Dll4, integrins (αvβ3, αvβ5, β1), SGK1-NDRG1 pathwaymyocardium.	Angiogenesis, reperfusion repair; integrin-mediated directional migration, vessel formation	73-81
Stem Cells (MSCs, CPCs, Sca-1+, Nanog+, hPFCs)	Migrate to infarcted myocardium post-MI in response to chemotactic signals	SDF-1/CXCR4, HGF/c-Met axis, HIF-1α, ICAM-1/VLA-4, LFA-1; IL-6, TNF-α affect migration/survival	Tissue repair, angiogenesis, immunoregulation, enhanced by gene editing, chemokine boosting, biomaterials	41, 82-89
Dendritic Cells (DCs)	Migrate from blood to infarct zone post-MI, then to lymph nodes	CCR2/CCL2, GM-CSF, HSPs, HMGB1; subtype-specific (e.g., cDC1, cDC2, pDC)	Antigen presentation, inflammation regulation, fibrosis resolution; subtype-dependent immune modulation	91-97
Pericytes	Actively migrate to infarct sites during vascular remodeling	PDGF-BB/PDGFR-β, VEGF-A, TGF-β1; MMP2/3/9, integrins; inflammatory cytokines (TNF-α, IL-1β, IL-10)	Promote angiogenesis and vessel stabilization; can transdifferentiate into myofibroblasts, contributing to fibrosis	98-108

**Table 2:** Comparative mechanisms of cell migration in myocardial infarction and heart failure.

Cell Type	Migration in MI	Migration in HF	Key Differences	References
Neutrophils	Rapid early migration to infarct site (hours after MI)	Persistent low-grade infiltration	Acute response vs. chronic activation; MI resolves quicker	112,113
Monocytes/Macrophages	Early recruitment, M1→M2 switch aids healing	Continuous recruitment, often M1-dominant	MI has resolution phase; HF maintains chronic inflammation	114,115
Fibroblasts	Migrate early for scar formation	Continuous activation and migration	MI migration is reparative; HF leads to excessive fibrosis	116-118
Endothelial Cells	Angiogenesis post-MI	Impaired angiogenesis; EndMT prevalent	MI promotes new vessels; HF has vascular rarefaction	119,120
MSCs	Directed homing to injury site	Migration reduced by chronic inflammation	HF needs assistance (e.g., gene editing) to restore migration	84,121-124
T Cells	Less prominent migration	Active, persistent migration especially CD8+ and Tregs	HF has more immune involvement and imbalance	125,126
DCs	Migrate to infarct then lymphoid organs	Low-level, chronic tissue migration	HF has lingering antigen presentation and immune crosstalk	127
Pericytes	Aid angiogenesis and vessel stability	Migration reduced, may convert to pro-fibrotic cells	HF-related pericyte dysfunction leads to capillary loss	101-105

**Table 3:** A brief table of challenges and prospects regarding the migration of heart cells.

	Key Points
**Challenges**	
Local Microenvironment	Hypoxia and inflammation after injury impair cell survival and migration; HIF-1α activation disrupts stem cell function 172.
Cell-Cell Interactions	Complex interactions among fibroblasts, immune cells, and endothelial cells regulate migration; CD40/CD40L axis amplifies inflammation; fibroblasts produce VEGF 83, 85.
Migration Limitation/Dysregulation	Misguided or excessive migration leads to fibrosis and cardiac dysfunction; e.g., unbalanced fibroblast migration causes collagen accumulation 173.
Biomaterials & Scaffolds	Materials like PLLA have weak bioactivity and poorly mimic cardiac microenvironment; surface modification and functionalization are research priorities 174.
**Future Strategies**	
Mechanism Exploration	Targeting key pathways (e.g., SDF-1/CXCR4) via gene editing or drugs to enhance migration and therapeutic effects 166.
Targeted Therapies	Engineering MSCs to overexpress CXCR4 improves responsiveness to SDF-1 gradient and migration efficiency 167.
Scaffold Optimization	Use of nanofiber scaffolds enhances adhesion and guided migration; e.g., nano-coated LAA occluder promotes rapid endothelialization and reduces stroke risk 168, 169.
Cell Enhancement	Hypoxic preconditioning improves MSC migration and anti-inflammatory properties 170.
Multidisciplinary Approach	Combining biology, materials science, and 3D printing to build microenvironment-mimicking scaffolds and support regeneration 171.

## Data Availability

The datasets used and/or analysed during the current study are available from the corresponding author on reasonable request.
